# Why hibernate? Predator avoidance in the edible dormouse

**DOI:** 10.1007/s13364-022-00652-4

**Published:** 2022-10-06

**Authors:** Thomas Ruf, Claudia Bieber

**Affiliations:** Department of Interdisciplinary Life Sciences, Research Institute of Wildlife Ecology, University of Veterinary Medicine, Savoyenstrasse 1, 1160 Vienna, Austria

**Keywords:** Multiday torpor, Predation pressure, Environment, Cold, Food

## Abstract

We address the question of ultimate selective advantages of hibernation. Biologists generally seem to accept the notion that multiday torpor is primarily a response to adverse environmental conditions, namely cold climate and low food abundance. We closely examine hibernation, and its summer equivalent estivation, in the edible dormouse, *Glis glis.* We conclude that in this species, hibernation is not primarily driven by poor conditions. Dormice enter torpor with fat reserves in years that are unfavourable for reproduction but provide ample food supply for animals to sustain themselves and even gain body energy reserves. While staying in hibernacula below ground, hibernators have much higher chances of survival than during the active season. We think that dormice enter prolonged torpor predominantly to avoid predation, mainly nocturnal owls. Because estivation in summer is immediately followed by hibernation, this strategy requires a good body condition in terms of fat reserves. As dormice age, they encounter fewer occasions to reproduce when calorie-rich seeds are available late in the year, and phase advance the hibernation season. By early emergence from hibernation, the best territories can be occupied and the number of mates maximised. However, this advantage comes at the cost of increased predation pressure that is maximal in spring. We argue the predator avoidance is generally one of the primary reasons for hibernation, as increased perceived predation pressure leads to an enhanced torpor use. The edible dormouse may be just an example where this behaviour becomes most obvious, on the population level and across large areas.

## Introduction

“Small animals have a high metabolic rate to begin with, and a further increase may be too expensive when food is scarce or unavailable. The easy way out, and the only logical solution is to give up the struggle to keep warm and let the body temperature drop. This not only eliminates the increased cost of keeping warm, but cold tissues use less fuel and energy reserves last longer. This is, in essence, what hibernation is all about”. This is how Schmidt-Nielsen (Schmidt-Nielsen 1979) in his textbook explains hibernation, and indeed the temporal escape from cold and the scarcity of food are at the centre of arguments for hibernation and estivation of mammals. Why is it then, that edible dormice in certain years enter prolonged torpor (estivation) during early summer when the vegetation, that provides ample food, is still growing? To make matters even more confusing: why is it that only the heaviest, fattest animal begin to estivate then while dormice In poor condition stay above ground (Hoelzl et al. 2015)? This behaviour seems to contradict the common rationales for hibernation.

In short, dormice enter hibernation in early summer when they skip reproduction, which is the case on average every three years (Fig. 2 in Ruf and Bieber 2020a). Dormice estivate only when they are in good body condition, because estivation is immediately followed by hibernation. Thus, it requires staying below ground and living on body energy reserves, particularly fat, for almost 1 year (Dark 2005; Hoelzl et al. 2015). The main reason why dormice avoid activity in the canopy is predator avoidance, mainly that of owls. Low predation and high survival rates generally seem one of the major advantages of hibernation (Turbill et al. 2011). There is also experimental evidence showing that small mammals use torpor to lower their predation risk (Turbill and Stojanovski 2018).

In edible dormice, hibernation is an integral part of their reproductive strategy. Although dormice can live in areas with a very low seed-tree abundance, they respond strongly to year-to-year fluctuations in the mast seeding of deciduous trees. Reproduction is tightly coupled to the availability of high-caloric seeds, particularly of beech trees (Bieber 1998; Schlund et al. 2002; Fietz et al. 2005; Ruf et al. 2006; Vekhnik 2019). The proportion of successfully reproducing females is high in full mast years with virtually all trees seeding, average in intermediate mast years, when only a fraction of trees produce seeds, and entire populations of dormice can skip reproduction in mast failure years (Fietz et al. 2005; Ruf et al. 2006). However, that species skip a year of reproduction and strongly respond to pulsed resources is not restricted to hibernators (Yang et al. 2010; Bergeron et al. 2011).

In dormice, certain years are unfavourable for reproduction but fully sufficient to gain mass on foliage, berries, or arthropods (Santini 1978; Koppmann-Rumpf et al. 2003; Fietz et al. 2005; Ruf et al. 2006). In those years, dormice may enter estivation after just 2–3 weeks of activity in late spring (Hoelzl et al. 2015). Here, we review the biology of hibernation in this species both as a regular component of its life history and a possible response to environmental conditions, such as predator abundance.

### Fattening and growth

**Fig. 1:**
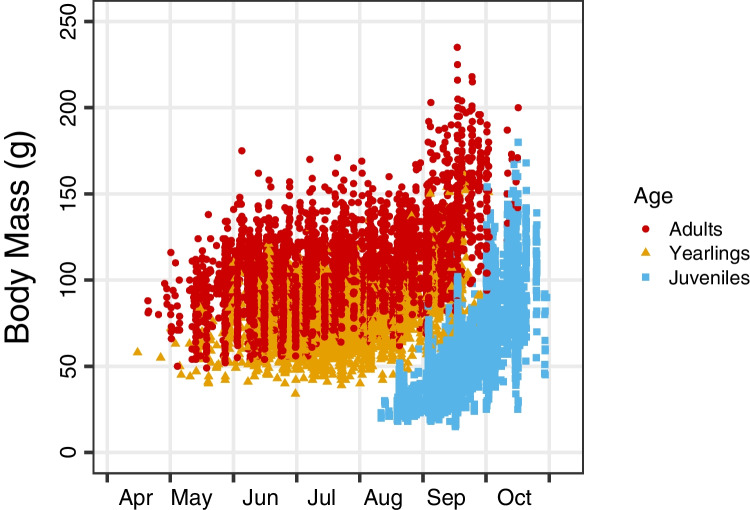
Body mass development over the active season in edible dormice. Body masses of adults, yearlings and juveniles in the Vienna woods at ~ 400 m a.s.l. In winter, animals hibernate below ground. Data from a capture-recapture study 2006–2020. Body masses are from both sexes, as no noticeable sex differences were found. Data from Bieber and Ruf (unpublished)

In the Vienna woods, adult and yearling dormice emerge in spring with a mean mass of 87 g. The last group to be encountered in traps or nest boxes are adult females, which is a proxy of emergence, are seen up to early July. This pattern is very similar to a site in Germany with a similar climate (Bieber and Ruf 2004). Dormice enter hibernation in autumn (after October 1) with an average mass of 154 g (Fig. 1). This corresponds to a 77% mass gain, and there is no notable size dimorphism between sexes. Similar mass gains over summer, sometimes with intermediate losses during reproduction, have been also observed at different sites in Germany (Bieber 1998; Schlund et al. 2002). Outside the distribution range of beech, e.g., in Lithuania, the seasonal weight gains are somewhat smaller (98–128 g, Juškaitis and Augutė, 2015). In England, on the other hand, a substantial fraction of animals even reach a pre-hibernation mass of > 300 g (Morris and Morris 2010). This fat gain is not unusual among hibernators, which often double their body mass during summer. Increases in body mass represent a programmed increase in fat deposition and, in certain species, of fat-free dry mass (Dark 2005). In the edible dormouse, a greater body mass seems to be entirely due to white adipose tissue deposition (Schaefer et al. 1976), and autopsies indicate the fat deposition is mostly subcutaneous and intraperitoneal. However, ultrasonography imaging revealed that the content of fat in the liver, distributed in an unexpected pattern of discrete focal areas, visibly increased at the end of the active season. This was accompanied by a significant increase in the transverse liver diameter of edible dormice (Bieber et al. 2011).

Experimental food self-selection trials showed that this body mass increase is predominantly caused by hyperphagia, first causing a large (up to tenfold) increase in carbohydrate uptake followed by a surge in lipid uptake (Jastroch et al. 2016). In natural environments, beech seeds or acorn would be a natural food source rich in lipids (Bieber and Ruf 2004). Like in other hibernators, the concentration of the gut-produced orexigenic hormone ghrelin probably gradually increases in dormice during summer to reach high values at the autumnal-hyperphagic period (Florant and Healy 2012). Peripheral injections of ghrelin cause the increase in food intake at all seasons, even in aphagic hibernators at the start of hibernation (Healy et al. 2011). However, food intake in hibernators is in fact controlled by a multitude of signals. Besides ghrelin, changes in circulating leptin and insulin, as well as in nutrients (glucose and free fatty acids), and cellular enzymes such as AMP-activated protein kinase (AMPK) determine the activity of neurons involved in the food intake pathway. The complexity of this system is underlined by the fact that during hyperphagia more than 900 genes can be differentially expressed in white adipose tissue (Jansen et al. 2019). Importantly, during the fattening phase, hibernators become temporarily insensitive towards leptin, the hormone that normally signals white fat content and limits lipid uptake and deposition. In hibernators, leptin can be temporarily disassociated from adiposity (Kronfeld-Schor et al. 2000; Florant and Healy 2012; Jastroch et al. 2016).

However, changes of body mass are not necessarily a consequence of increased food intake alone. In certain hibernators, but not in others, resting metabolic rate at euthermia can decrease in late summer, several weeks before food intake declines, thereby enhancing late lipid deposition by shifting the energy balance (Dark 2005; Sheriff et al. 2013). It remains to be seen if edible dormice also use this mechanism to aid pre-hibernation fattening.

Dormice give birth late in the active season, mostly in August. Juvenile dormice increase body mass extremely fast, which includes both structural growth and fattening (Fig. 1). At the Austrian study site, juveniles grow from ~ 30 g prior to September 1, around weaning, to an average of 86 g right before hibernation, i.e., after October 1. This is equal to a mass gain of 280% in 2–3 months. Data from other species with one litter per year suggest that in hibernators birth weights are at, or slightly below, the values expected from the allometric curve for mammals (Peters 1993; Bieber and Ruf 2004). Weaning weights, however, are typically higher than expected for mammals of that size (Millar 1977), indicating that growth rates in hibernators may be generally high. In the closely related species, *Eliomys quercinus*, a hibernator that may have several litters per year, growth and fatting of juveniles are supported by their use of torpor during summer. *G. glis* is also capable of short torpor, but it still has to be evaluated if this behaviour helps to gain mass in this species.

A humoral pathway that can facilitate growth are high glucocorticoid levels. It has been demonstrated that female red squirrels provisioned with cortisol have faster growing pups, and that the growth of young is sped up by maternal effects of high glucocorticoids, especially when they experienced a high population density (Dantzer et al. 2013). High amounts of cortisol metabolites were indeed found in reproducing dormice, and only in reproductive years (Cornils et al. 2018; Havenstein et al. 2021). Partly, high cortisol levels in dormice may reflect their response to stressors caused by reproductive activity, such as competition for mates and exposure to predators during high foraging periods (Cornils et al. 2018; Havenstein et al. 2021). However, a major cause for high glucocorticoid levels, which are more than twice as high in females than in male dormice (Havenstein et al. 2021), their beneficial effect is probably increased growth of the young, although the exact energetic source of which still has to be determined (Dantzer et al. 2013). The mass gain of both young and adults seems to be a prerequisite for hibernation, since it is accompanied, as in many hibernators, by the complete cessation of foraging, no matter what the environmental food situation.

### Hibernation and estivation timing

Dormice normally commence hibernation in September to November, first are males followed by females. In both sexes, hibernation onset is delayed by reproductive investment by 2–3 weeks (Ruf and Bieber 2020a). These delays occur among females that actually give birth to young and males that develop large testes and become sexually competent. The last class to enter hibernation is juveniles (Fig. 1). Dormice hibernate close to their summer nests, 90% of the hibernacula are less than 400 m from the “home” nest box (Trout et al. 2018). *G. glis* usually hibernates below ground in self-dug underground chambers, typically alone or accompanied by one or two conspecifics (Morris and Hoodless 1992; Jurczyszyn 2007; Trout et al. 2018).

**Fig. 2:**
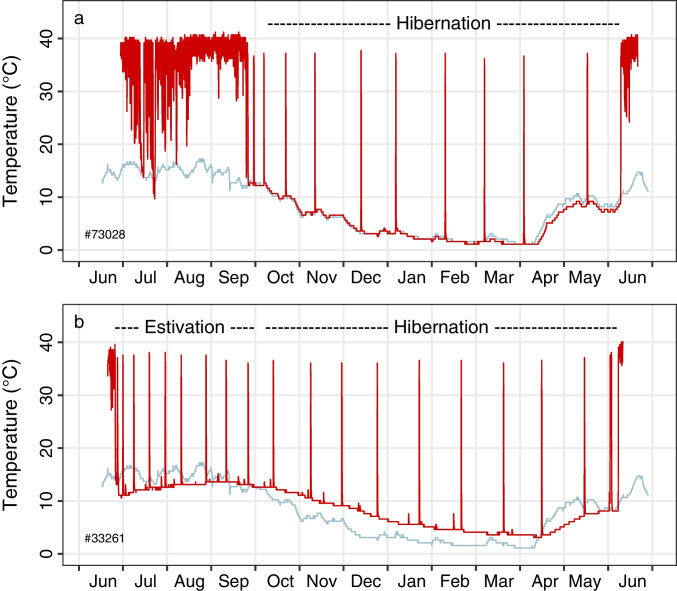
**a** Hibernation as well as **b** estivation and hibernation in non-reproductive years in edible dormice. Prior to hibernation the animal in Fig. 2a (#73,028) displays many bouts of short torpor, particularly early in the summer season (July to early August). The dormouse in Fig. 2a also shows two episodes of multiday torpor in July. The dormouse in Fig. 2b (#33,261) enters estivation (hibernation in summer) already in June, data from Hoelzl et al. (2015). Light blue lines show soil temperature in the middle of the study area at a depth of 60 cm, the approximate depth of burrows that are relatively shallow (30–80 cm: Ruf and Bieber 2020a). Apparently, the soil temperature was measured closer to the hibernaculum in Fig. 2a than in 2b. Negative gradients between body and environment in Fig. 2b imply a well-insulated hibernaculum. The red lines show body temperature

Hibernation, i.e., multiday torpor in edible dormice in Austria normally starts after one or two initial short deep torpor bouts in September/October (Fig. 2a). It lasts on average until June, so mean hibernation duration is slightly more than 8 months in this population. This duration of 7–8 months seems to be quite typical for the central and northern edge of its distribution (Lebl et al. 2011; Juškaitis and Augutė, 2015). The shortest active season and longest hibernation of dormice may be that in the Kazan region of Russia (Rossolimo et al. 2001). On the southern edge of the range, in central Italy and Sicily, the hibernation season of dormice may last just 6 months (from November/December until late April to May, Santini 1978; Milazzo et al. 2003). It seems, however, that most estimates of hibernation duration are based on the long-term absence of animals from nest boxes (which generally is a reliable measurement of occupancy; Bieber and Ruf 2009), while the only long-term body temperature records in free-living dormice are those from the Austrian population (e.g., Hoelzl et al. 2015).

These records show that, in most cases, animals display numerous short torpor bouts during the summer season in non-reproductive years in nest boxes, typically rewarming to euthermia (~ 37–40 °C) and activity during the night (Fig. 2a). Dormice may also show prolonged episodes of torpor in underground burrows (Hoelzl et al. 2015). This behaviour is most prominent in the early active season. In years of sexual activity, hibernation onset is also only in autumn, but short torpor early in the year is virtually absent (Bieber and Ruf 2009; Bieber et al. 2017). As mentioned above, in non-reproductive years, dormice alternatively may re-enter hibernation, also called estivation as it occurs in summer, only a few weeks after their emergence from the previous hibernation. This way, dormice may spent up to 11.4 months in continuous hibernation (Hoelzl et al. 2015). Estivation is restricted to the ~ 50% heaviest animals with large body fat reserves, albeit this dichotomy is not absolute (Hoelzl et al. 2015).

Entry into hibernation in *G. glis* is characterised by a rapid decline of heat production and metabolic rate, followed by a much slower decrease in body temperature (Wyss 1932; Wilz and Heldmaier 2000). The average metabolic rate reached in hibernation is 0.017 ml O_2_ g^−1^. This is a reduction to ~ 2% of basal metabolic rate. The minimum body temperature observed was 0.7 °C, and the temperature difference between body and environment was only 0.2 °C, on average, in deep hibernation (Wilz and Heldmaier 2000). Like most hibernators, dormice enter hibernation in a curled, ball-like position that minimises heat loss, and thermal conductance stays minimal and constant in torpor (Wilz and Heldmaier 2000). This underlines that metabolic reductions by hibernation are mainly achieved by lowering the heat loss gradient to the environment (Heldmaier and Ruf 1992). Metabolic decreases are not much supported by facilitated cooling, which would be expected if pure tissue temperature (Q10) effects were important. It seems that pure temperature (Q10) effects play only a negligible role and that the lowering of metabolic rate in hibernation is mainly due to an active downregulation (Nogueira de Sá and Chaui-Berlinck 2022). In this context, it should be noted the reducing respiration and metabolism in hibernation has several effects: not only does it minimise energy expenditure, it also helps the animals to remain motion, and largely odourless, which makes them difficult to detect for predators (Turbill et al. 2011; Ruf et al. 2012).

Both hibernation and estivation (Fig. 2a, b) are interrupted by periodic rewarming, also called arousals, to interbout euthermia. These arousals are typical for the vast majority of hibernators and were first described by Hall (1832) for bats, hedgehogs, and common dormice. It has been argued thar periodic arousals are beneficial for sleep, the immune system, or heart function. However, it still remains unclear why hibernators periodically rewarm from torpor, especially since these episodes are responsible for at least 70% of the energy expenditure during winter (Wang 1979; Strijkstra 1999). For a more thorough discussion of this topic, see Ruf et al. (2022). Both a comparison between hibernators and data from within a species (*E. quercinus*) show that the duration of torpor bouts is shortened if the metabolic rate during torpor is increased. This strongly points to an increasing metabolic imbalance during torpor, such as the depletion of a crucial substance, which is restored during interbout euthermia at high body temperature (Ruf and Geiser 2015; Ruf et al. 2021). The duration of torpor bouts in *G. glis* is the longest among hibernating rodents, only surpassed by a few species of bats. The maximum torpor bout duration in dormice is often above 6 weeks (Fig. 2a), the absolute maximum is 1278 h (7.6 weeks), and the average duration of interbout euthermia is 6.7 h (Hoelzl et al. 2015; Ruf and Geiser 2015).

The duration of torpor bouts is subject to evolution among hibernators. Torpor bout duration increases, and hence the frequency of costly arousals decreases, with increasing distance of the species distribution centre from the equator (Ruf and Geiser 2015). As mentioned above, the same tendency can be found within *G. glis*, i.e., a gradient in torpor bout duration between Lithuania and the Mediterranean (Rossolimo et al. 2001; Milazzo et al. 2003).

There are only two known environmental factors that affect torpor bout duration in hibernation. The first is dietary fatty acids. Increased dietary uptake of n-6 polyunsaturated fatty acids, particularly of linoleic acid (LA, C18:2 n-6), enables animals to reach lower body temperatures, lengthens torpor bout duration, and results in lower energy expenditure during hibernation (Arnold et al. 2015; Giroud et al. 2018). In fact, providing LA in the diet can more than double torpor bout duration (Munro and Thomas 2004; Ruf and Arnold 2008). Dormice of the sister clade *E. quercinus* even delay hibernation and remodel their membranes if fed a LA deficient diet (Giroud et al. 2018). However, LA apparently is no limiting environmental factor for edible dormice, which over the summer season increasingly digest seeds very rich in LA, and deposit it in white fat (Fietz et al. 2005). It would be interesting to study torpor bout duration and hibernation energy expenditure in areas that lack LA-rich seeds.

The second extrinsic factor that is known to influence torpor bout duration is ambient temperature. Increasing environmental temperatures shorten torpor bout length and increase arousal frequency both in hibernators in general and in edible dormice (Bieber and Ruf 2009; Geiser 2021). There is a single linear relationship between soil temperature and bout duration, which approximately doubles as ambient temperature decreases by 10 °C (Bieber and Ruf 2009). Hibernators respond even to the < 3 °C fluctuations of a cooling chamber (Ruf et al. 2021). Thus, rapidly globally rising temperatures, if the rise is too fast to allow evolutionary adaptation (Hoffmann and Sgrò 2011), expectedly will lead to an increase in rewarming frequency and energy depletion during hibernation. Hence, climate change poses a severe threat for *G. glis* and other hibernators.

Estivation in *G. glis* occurs in animals without severe cold load during summer (Fig. 2b). In fact, a good body condition in terms of ample fat reserves is even a prerequisite for this behaviour (Bieber and Ruf 2009; Hoelzl et al. 2015). Hence, at least in this species, energy savings seem not the primary goal of retreating to underground burrows during summer.

In this regard, *G. glis* appears to differ from other mammals that undergo estivation mainly to endure periods of severe heat and drought (Geiser 2010). Well-known examples are north American and Australian species that may enter torpor in summer (Bartholomew and Cade 1957; Bartholomew and MacMillen 1961; Bartholomew and Hudson 1962; Brice et al. 2002; Turner et al. 2012). Data on edible dormouse estivation underline the functional uniformity of hibernation and estivation. The frequency and duration, for example, of arousals follow identical dependencies on burrow temperature in summer and winter (Bieber and Ruf 2009). The motives for employing estivation may however differ between species.

**Fig. 3:**
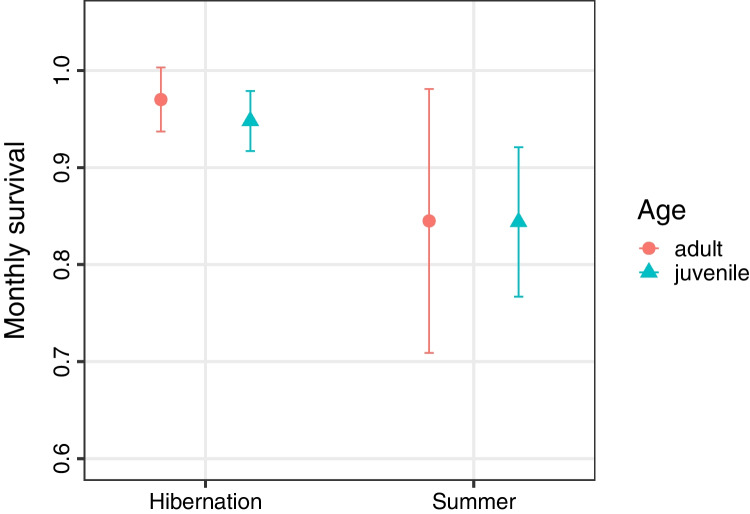
Monthly survival rates in 19 species of hibernators are significantly higher (*P* < 0.05) during hibernation vs. the remainder of the year. *N* = 38 populations (adults) and *N* = 8 populations (juveniles), data from Turbill et al. (2011)

Arguably, edible dormice and several other hibernators use hibernation below ground in non-reproductive years to escape their predators, which include both mammals and nocturnal birds of prey (Kryštufek 2010). A comprehensive comparative study on hibernators in general revealed that monthly survival is significantly higher during hibernation than during the active season (Turbill et al. 2011; Fig. 3). Since predation is the main cause of mortality in small rodents, this is likely the main source of these differences.

As mentioned before, predator avoidance is augmented by the fact that cold, torpid mammals hardly emit any odours. In fact, Brown (1970) introduced hungry weasels on several occasions into a room containing hibernating jumping mice, and the predators were unable to locate the hidden, but accessible mice. Thus, reports of predators, such as American badgers, specialising on hunting hibernators seem to be the exception, rather than the rule (Michener 2004). Hibernation as an escape from predators is particularly pronounced in the edible dormouse. Comparing dormouse populations from five European countries, Lebl et al. (2011) found that survival rates were close to 100% in hibernation, and were lowest in early summer in all populations (monthly survival ~ 80% or below). It is at this time of the year in spring that avian and mammalian predators reproduce themselves and show increased foraging activity. This work, especially the seminal paper by Turbill and colleagues (2011) has led to a new view: Hibernation is no longer regarded as merely a response to harsh environmental conditions but also serves to minimise predation. In edible dormice, entire summer seasons are spent in estivation in years of reproduction skipping, and long-term torpor has become an integral part of their life history. Hibernators are generally long-lived, and have long generation times (Turbill et al. 2011). The maximum lifespan of edible dormice in England, Germany, and Austria is 13–14 years (Trout et al. 2015; Havenstein et al. 2021, and C. Bieber pers. comm.). An important prerequisite for this high longevity in a 100-g rodent is low extrinsic mortality, for instance by a low predation rate on younger adults (Kirkwood 1977). Only if extrinsic mortality is low, it pays to invest in cellular maintenance and high longevity. Apparently, one important factor for this decreased mortality is the repeated absence from aboveground, and estivation instead, of *G. glis*.

Age has a profound effect on hibernation, primarily because in dormice there is an intricate relationship between hibernation and reproduction. As dormice grow older, the probability to reproduce increases as there are diminishing chances for future reproduction (Bieber et al. 2018; Ruf and Bieber 2020a). In other words, old dormice cannot afford to ‘sit tight’ forever until environmental conditions are optimal for reproduction. With increasing age, edible dormice therefore phase advance the entire hibernation season, with a most pronounced forward-shift of spring emergence. By coming out of hibernation, early females will have access to the best territories (Bieber et al. 2018). Despite their use of huddling, mainly with related individuals, dormice defend foraging territories (e.g. Cornils et al. 2017; Ruf and Bieber 2020b). An earlier onset of reproduction generally gives offspring a better chance to survive the following winter, which is also true for dormice (Pilastro et al. 1994). The timing of emergence from hibernation in spring is also sex-specific (Vietinghoff-Riesch 1960; Bieber 1998; Schlund et al. 2002). In edible dormice, as in many hibernators, males emerge before females (Michener 1983; Körtner and Geiser 1998; Blumstein 2009; Lane et al. 2011). An early emergence of males is thought to enhance their reproductive success by maximising the number of potential mates available. However, the early termination of hibernation will expose both sexes to additional predation pressure, which is particularly high in spring (Lebl et al. 2011). Together, this evidence shows that hibernation is a flexible life history trait, with its duration and timing depending on an animals’ age, body condition, and residual reproductive value.

### Trade-offs and seasonal clocks

The lowering of body temperature to save energy as well as the escape from predation during torpor comes at a cost. This can be most easily seen by the fact that short torpor is almost completely restricted to non-mast, non-reproductive years (Bieber et al. 2017). It has long been known that torpor is largely incompatible with reproduction in rodents (Fietz et al. 2004;; but see McAllan and Geiser 2014). In males, high testosterone levels prevent torpor and high body temperatures are required for spermatogenesis (e.g., Barnes et al. 1987). Only in reproductive failure years dormice may save energy by lowering body temperature during the day and return to activity during the night (Hoelzl et al. 2015; Bieber et al. 2017). Occasionally, dormice show torpor episodes lasting several days during cold spells early in the summer season (Hoelzl et al. 2015). Dormice use short torpor predominantly in the early active season but hardly in its second half, just prior to hibernation (Fig. 2). This period of pre-hibernation fattening is associated with intense, energy-consuming foraging leading to body temperatures above 40 °C for several hours per night (Bieber et al. 2017). Possibly, torpor is avoided during this time because high rates of digestion of food and fat deposition require high body temperatures. It has been suggested that the use of torpor generally has a negative impact on activity because the reduction of energy expenditure during torpor allows animals to minimise foraging (Ruf and Heldmaier 2000; Turbill and Stojanovski 2018). When reproductive activity prevents torpor, but also in non-reproductive years, dormice use an alternative avenue of energy savings: they utilise huddling and communal nesting of up to 16 animals at a time (Ruf and Bieber 2020b). Social thermoregulation is particularly used at low ambient temperatures during summer and is employed mainly by small dormice, often by yearlings, and frequently by related individuals (Ruf and Bieber 2020b).

**Fig. 4:**
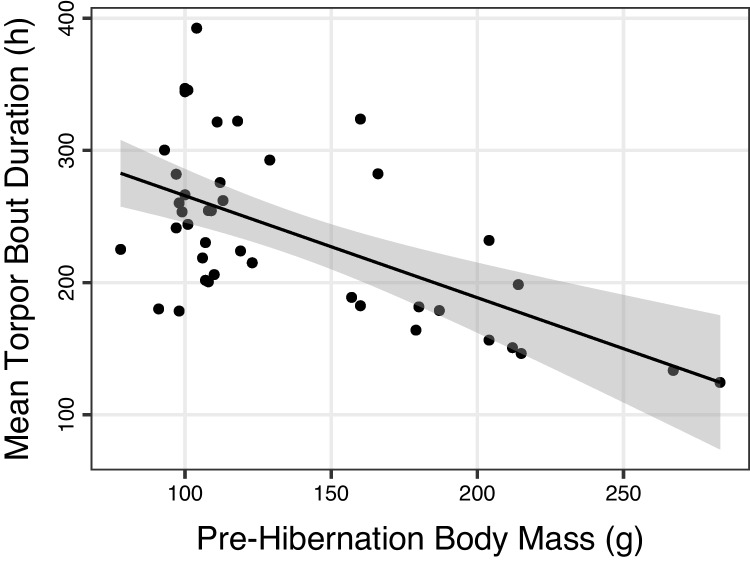
The mean duration of torpor bouts during hibernation as a function of body mass prior to hibernation inedible dormice. Particularly, fat animals minimise hibernation and show relatively short torpor bouts, data from Bieber et al. (2014). Animals were kept in large outdoor enclosures and hibernated in the soil

An indication of negative, potentially risky effects of deep torpor comes from a closer analysis of hibernation (Bieber et al. 2014). This analysis shows that fat dormice with large white adipose tissue depots avoid deep torpor have shorter mean torpor bout length and rewarm more frequently (Bieber et al. 2014; Fig. 4). Torpor avoidance, if possible, may be a general phenomenon since almost identical effects of increasing body mass were obtained from woodchucks (Zervanos et al. 2014). Also, chipmunks avoid torpor when ample food is available (Humphries et al. 2003). The maintenance, for example, of a continued cardiac function at a body temperature around 0 °C may be particularly challenging and is avoided whenever possible (Ruf and Arnold 2008; Bieber et al. 2014; Giroud et al. 2018).

Another example for trade-offs involved in hibernation is the accompanying shortening of telomeres, a marker of somatic maintenance and damage. Together with the telomere-associated protein, telomeres prevent the degradation of linear DNA, but shorten with every cell division—because of the end replication problem in mitosis (Blackburn 1991; Blasco 2007). In addition to the shortening during cell proliferation, oxidative stress has a strong effect on telomere erosion (Proctor and Kirkwood 2002; von Zglinicki 2002). High oxidative stress during rewarming from deep torpor (Buzadžić et al. 1990; Carey et al. 2000; Orr et al. 2009) is probably the reason why telomeres in dormice are shortened more with an increasing number of arousals (Hoelzl et al. 2016a). This negative effect of hibernation is surprising, because hibernators in general, and dormice in particular invest in somatic maintenance and are very long-lived. It turns out that *G. glis* is highly unusual because animals older than ~ 5 years actually prolong their telomeres again (Hoelzl et al. 2016b), perhaps to prepare for telomere loss due to oxidative stress during reproduction. The net effect on telomeres is shortening in younger and elongation in older dormice (Hoelzl et al. 2016b).

Telomere elongation takes place not only in spring after hibernation (Hoelzl et al. 2016a) but even the last third of the hibernation season itself (Nowack et al. 2019). This finding points to an endogenous seasonal rhythm of telomere control, just like of hibernation itself, which for example is terminated without any photoperiod or temperature cues (see Fig. 1 in Hoelzl et al. 2015). In many species, hibernation is controlled by an endogenous circannual clock that is normally entrained by photoperiod (Pengelley and Fisher 1957; e.g., Fisher 1964). Ground squirrels kept in constant conditions showed a cycle of about 11 months (Davis 1976). We know now that the seasonal control of hibernation involves a cell population called tanycytes adjacent to the third ventricle of the brain. Tanycytes have been identified as major players in the seasonal control of energetic states in mammals; however, the exact mechanism of hibernation control is currently unknown (review in Jastroch et al. 2016).

It has been suggested that *G. glis* requires normal seasonal fluctuations of temperature to show a circannual cycle of hibernation, which is why this species has been called “thermoperiodic” (Jallageas et al. 1989). Mrosovsky (1977) found that body mass and hibernation cycles had a mean length of only 53 days (range 22–85 days) at 22 °C, whereas dormice in a cold room at 5 °C had a mean cycle length of 162 days (range 28–425 days). Hence, it seems that ambient temperature indeed affects annual cycles, but the mechanism causing circannual hibernation rhythms in *G. glis* is less than clear. However, the above conclusions were based on experiments in the laboratory with animals kept in standard cages. Notably, edible dormice are extremely reluctant to display normal torpor if they do not have access to self-dug hibernacula in the soil that may convey relative safety from predation. Thus, our understanding of genuine hibernation cycles in *G. glis* may require experiments that combine constant photoperiod or temperature with conditions of simulated natural hibernacula.

## Conclusions

Dormice clearly use estivation and hibernation not primarily to avoid cold environments and a scarcity of food, but to avoid predation by remaining in hibernacula whenever possible. This becomes evident when they enter long torpor during a time when there is ample food to survive but predators are active. As a consequence, edible dormice spend up to three quarters of their life in hibernation, hidden below ground. In dormice, this extreme hibernation is linked to the pattern of reproduction, and absent in areas where reliably fruiting beech are not the dominating tree (Vekhnik 2017, 2019). This raises the question whether *G. glis* is unique in this respect or reflects a widespread pattern. Among hibernators, a close link to pulsed resources is only known from Eastern chipmunks in North America. Strikingly, chipmunks were found to interrupt aboveground activity for 9–11 months when mast was not available (Munro et al. 2008). However, while hibernation in response to pulsed good availability seems rare, it is quite possible that this link only becomes obvious on a population level, when all or most individuals remain absent in certain years. Given that increasing the perceived predation pressure enhances the use of torpor (Turbill and Stojanovski 2018), it is likely that other hibernators individually, and locally, trade reproduction against safety from predators. Possibly, this trade-off is as important in causing hibernation as the harshness of environments.
